# Luteolin, quercetin, genistein and quercetagetin inhibit the effects of lipopolysaccharide obtained from *Porphyromonas gingivalis* in H9c2 cardiomyoblasts

**DOI:** 10.1186/s11658-017-0047-z

**Published:** 2017-09-04

**Authors:** Gloria Gutiérrez-Venegas, Alfredo Torras-Ceballos, Juan Arturo Gómez-Mora, Berenice Fernández-Rojas

**Affiliations:** Laboratorio de Bioquímica de la División de Estudios de Posgrado de la Facultad de Odontología, Universidad Nacional Autónoma de México Ciudad Universitaria, 04510 México DF, Mexico

**Keywords:** Cardiomyoblasts, Flavonoids, Mitogen-activated protein kinase, Lipopolysaccharide

## Abstract

**Background:**

One of the microorganisms from dental plaque associated with severe inflammatory responses in infectious endocarditis is *Porphyromonas gingivalis*. It is a Gram-negative bacteria harvested from chronic periodontitis patients. Lipopolysaccharide (LPS) obtained from *P. gingivalis* promotes the expressions of interleukin-1 (IL-1), IL-6 and tumor necrosis factor alpha (TNF-α). Flavonoids are thought to participate in processes that control inflammation, such as the expression of cyclooxygenase-2 (COX-2).

**Methods:**

We investigated the effects of luteolin, quercetin, genistein and quercetagetin on cardiomyoblasts treated with LPS alone or in combination with following inhibitors p38 (SB203580), ERK (PD98059), JNK (SP600125) and PKC (Calphostin C) for 1 h. The kinase activation and COX-2 expression levels were determined at the gene and protein levels.

**Results:**

These flavonoids are considered to inhibit the activation of mitogen-activated protein kinase (MAPK) and the degradation of inhibitor of kappa B-alpha (IκB-α). They also play a role in COX-2 expression.

**Conclusion:**

We conclude that the tested flavonoids inhibit inflammatory responses induced by LPS in H9c2 cells.

## Background

Poor dental hygiene favors bacterial inflow into the bloodstream of the mouth. Such bacteria can form colonies in the heart valves, causing a local infection called infective endocarditis [1–5]. The significance of bacteremia caused by dental extraction has not yet been fully characterized, but antibiotic prophylaxis has been widely used in its prevention [6].

*P. gingivalis* is found in dental plaque and associated with chronic periodontitis. LPS obtained from aforementioned bacteria induces pro-inflammatory processes and initiates a wide range of events that trigger destructive developments, but it also induces continual secretion of several cytokines, such as TNF-α [7], COX-2 [8], interleukin-1-beta (IL-1β) [8] and IL-6 [9], which are determinant molecules for tissue destruction.

Toll-like receptor 4 (TLR4) is a primary receptor for LPS [10]. LPS activates transcription for nuclear factor kappa-light chain-enhancer of activated B cells (NFκB). This transcriptional activity is associated with the expression of genes dependent on this factor and requires the stimulation of MAPK [11–13].

**Fig. 1:**
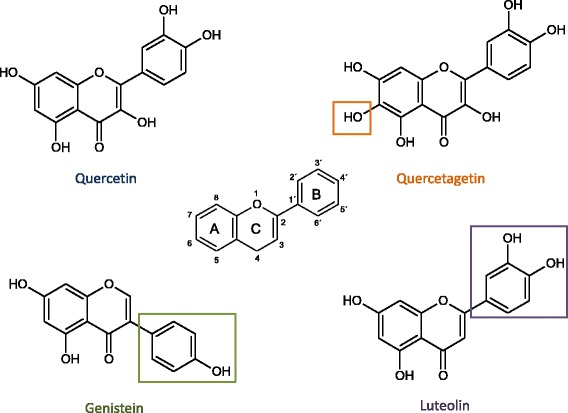
The chemical structures of quercetin, quercetagetin, genistein and luteolin. The central structure is that of a flavone. It has two phenyl rings (A and B) joined by a pyran ring (C). The boxes frame the substituents of their structure with respect to the flavonoid quercetin

Natural polyphenols found in different plants including vegetables and fruits. It has been claimed these molecules are able to inhibit inflammation in different cells. Figure 1 shows the molecular structure of the flavonoids employed: luteolin, quercetin, genistein and quercetagetin.

Luteolin (3′,4′,5,7 tetrahydroxyflavone) is an important flavone present in broccoli, pepper, thyme and celery and naturally found in a glycosylated form. Various studies showed that it possesses anti-inflammatory activity due to kinase inhibition and inhibition of pro-inflammatory substances [14–16] and that it prevents oxidative stress-induced cardiomyoblast apoptosis [17]. In LPS-stimulated murine macrophages of the RAW 264.7 cell line, luteolin inhibited TNF-α and IL-6 release, tyrosine phosphorylation, NF-κB-mediated gene expression, and protein kinase B (AKT) phosphorylation [18]. Luteolin was more effective than luteolin 7-glucoside, quercetin or genisteineriodictyol, with a 50% inhibitory concentration (IC_50_) value lesser than 1 μM for TNF-α release [19].

Quercetin (3,3′,4′,5,7-pentahydroxyflavanone) is a flavonol that displays protective effects against oxidative stress-induced cardiomyoblast apoptosis [20–22]. Quercetin inhibits LPS-induced TNF-α production in macrophages [23] in additon to LPS-induced IL-8 production in A549 lung cells [24]. Quercetin can inhibit LPS-induced mRNA levels of COX-2, reducing apoptotic neuronal cell death caused by microglial activation [25].

Genistein is an isoflavone (4′,5,7-trihydroxyisoflavone,5,7-dihydroxy-3-(4-hydroxyphenyl)-4-H-1-benzopyran-4-one) that diminishes the production of nitric oxide (NO) and prostaglandin E3 in BV2 microglia stimulated with LPS [26]. Quercetagetin (6-hydroxyquercetin) inhibits LPS-mediated COX-2 induction in human gingival fibroblasts [27].

We studied the regulatory roles of luteolin, genistein, quercetin and quercetagetin in the signaling pathways stimulated by LPS treatment in cardiomyoblasts. We found that the studied flavonoids reduced the phosphorylation of LPS-stimulated MAPK and COX-2 expression in a similar fashion.

## Methods

### Materials

The H9c2 cell line was obtained from the American Type Culture Collection (ATCC CRL-1446). Dulbecco’s modified Eagle’s medium (DMEM), fetal bovine serum (FBS), penicillin, streptomycin, trypan blue and Super Script One-Step Reverse transcription-polymerase chain reaction (RT-PCR kits were purchased from Invitrogen (Carslab, CA, USA). Luteolin, genistein, quercetin, quercetagetin, phenylmethylsulfonyl fluoride, sodium dodecyl sulfate (SDS) and ethylene diamine tetraacetic acid (EDTA) tetrazolium salt were obtained from Sigma Aldrich (St. Louis Mo, USA). LPS obtained from *P. gingivalis* (InVivo Gen, San Diego California USA), antibodies against p38, γ-tubulin, p50, phospho-extracellular signal-regulated kinase (ERK Thr 202/Tyr 204), phospho-p38 (Tyr 182), phospho-AKT (with the C-terminal at Ser 43), COX-2, IκBα, IκBβ and luminol reagent were purchased from Santa Cruz Biotechnology (Santa Cruz, CA, USA).

### Cell culture

Cells were grown in DMEM with 10% FBS, 100 U/ml penicillin, 100 μg/ml streptomycin and 2 mM L-glutamine, incubated at 37 °C in a humidified atmosphere with 5% CO_2_.

### Cell treatment

**Fig. 2:**
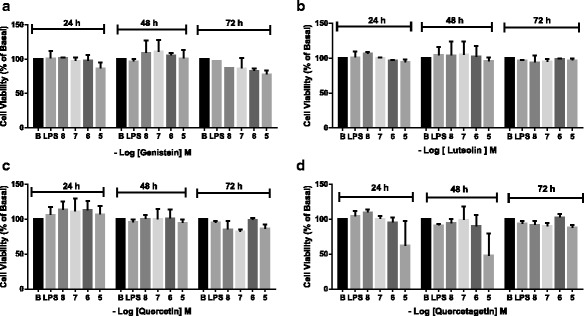
Flavonoid effect on cell viability. Cell viability in the presence of genistein (**a**), luteolin (**b**), quercetin (**c**) and quercetagetin (**d**) was evaluated using the MTT assay. H9c2 cells were treated with flavonoid (10 nM −10 μM) for 24, 48 and 72 h. Quercetagetin reduced cell viability at the highest concentration at 24 and 48 h without significant difference. No other flavonoid modified cell viability. The results are expressed as means ± SEM from at least three independent assays

H9c2 cells were grown overnight on 6-well plates at a concentration of 20,000 cells/well. After the culture medium was replaced with 2% SBF medium, cells were treated with flavonoids (10 μM) for 1 h and after that with LPS (1 μg/ml) as indicated in Fig. 2a–d. Experiments were also performed with specific inhibitors against p38 MAPK (50 μM SB203598); MAPK kinase (MEK) 1/2 (10 μM PD98059); c-Jun N-terminal kinases (JNK, 10 μM SP600125) or protein kinase C (PKC, 1 μM Calphostin C) for 1 h before LPS exposure. The negative control was cells either untreated or treated with dimethyl sulfoxide (DMSO; Sigma-Aldrich, St Louis MO, USA) at a concentration equal to that found in the flavonoids samples (0.15% *v*/v). After 4 h treatment, medium samples were collected for protein expression analysis and after 24 h, for cytokine measurements. Proteins were extracted from cells after a single wash with phosphate-buffered saline (PBS) and the whole cell extract was obtained in lysis buffer. All experiments were repeated at least three times.

### Cell viability assays

Cell viability was measured using the 3-(4,5-dimethyldiazol-2-yl)-2,5-diphenyltetrazolium bromide (MTT) assay, which determines mitochondrial activity [28]. Cells (4 × 10^5^) were plated onto 96-well plates, stained for viability with trypan blue, and counted in a Newbauer chamber. After overnight culture, the cells were treated with varying doses of flavonoids (10 nM – 10 μM) or LPS for 24, 48 or 72 h at 37 °C in a 5% CO_2_ atmosphere. When incubation was completed, 50 μl of MTT (5 mg/ml) solution in PBS was added to the wells. Plates were then incubated for 4 h to dissolve purple formazan crystals, 150 μl of DMSO was added and the plates were shaken for 30 min. Absorbance was measured at 540 nm using a microplate reader.

### Western blotting analysis

H9c2 cells (1 × 10^4^/well) were grown in 6-well plates (Corning, N.Y., U.S.A.**)**. Briefly, cells were treated with flavonoids (10 μM) for 1 h prior to treatment with LPS (1 μg/ml). Thereafter, the medium was aspirated, the cells were washed twice with PBS, and then put in 50 μl of cold lysis buffer consisting of 0.05 m Tris-HCl (pH 7.4), 0.15 M NaCl, 1% Nonidet P-40, 0.5 M phenylmethylsulfonyl fluoride (PMSF), 10 μg/ml leupeptin, 0.4 mM sodium orthovanadate, 10 mM sodium fluoride and 10 mM sodium pyrophosphate (all from Sigma Chemical Co., St. Louis, MO, USA).

Cells were scraped off, and the lysate was transferred to a micro-centrifuge tube, to be pulse-sonicated (1 s × 30) on ice. The protein concentration was measured using the Bradford protein assay. All samples were separated on a 10% gel for SDS-PAGE. Proteins were transferred onto polyvinylidene difluoride (PVDF) membranes by electro-blotting (Amersham, Piscataway NJ, USA) using the semi-dry method. Membranes were blocked in a 5% non-fat milk solution for 1 h at room temperature before being probed with primary antibodies overnight at 4 °C. Primary antibody dilutions were 1:20,000 with tubulin, which was used as an internal loading control. Horseradish peroxidase-linked anti-rabbit secondary antibody (1:10,000) was used to detect the primary antibody of COX-2, while an anti-mouse secondary antibody (1:25,000), was used to detect phosphorylated proteins. Immunoreactive bands were developed using a chemiluminescent substrate (Santa Cruz Biotechnology, Inc.). The autoradiograph was obtained with a 5-min exposure. Three different experiments were carried out for each figure. Equal loading of blots was demonstrated by stripping blots and re-probing with antibodies for total α-tubulin.

### Statistical analysis

Statistical analysis of densitometric data was performed by determining the integrated optical density (OD) of each sample and using analysis of variance (ANOVA). Any difference between the two groups with a value of p < 0.05 was considered significant.

## Results

### Chemical structure of flavonoids

Luteolin was found to have 5, 7, 3′ and 4′ substituted hydroxyls and a double bond presence at carbons 2 and 3, which are responsible for their multiple pharmacological effects. Genistein is a natural isoflavone compound with hydroxyls groups at positions 5, 7 and 4′. It is a specific and potent tyrosine kinase inhibitor that exerts anti-oxidative activity. Quercetin is a flavonol that has an OH group attached at positions 3, 5, 7, 3′, and 4′. Quercetagetin is a flavonol compound that has 3, 5, 6, 7, 3′ and 4′ –OH groups based on the molecular structure of the flavone backbone (2-phenyl-1,4-benzopyrone). It has many effects, including antifungal, antibacterial and antioxidant activities.

### Flavonoid effect on cell viability

We examined the cytotoxicity of luteolin, genistein, quercetin and quercetagetin over a wide concentration range and different periods using an MTT assay on H9c2 cells. Cells were treated with flavonoid (10 nM – 10 μM) and incubated for 24, 48 and 72 h. At the concentrations evaluated, flavonoid treatment had no cytotoxic effect. However, at 10 μM for 24 or 48 h, quercetagetin reduced H9c2 viability. Low concentrations of quercetagetin (less than 10 μM) did not have a significant effect on the viability or survival of the H9c2 cells (Fig. 2). Thus, we used 10 μM concentrations of flavonoids in subsequent experiments (Fig. 2a–d).

### Effects of LPS on the activation of MAPK in H9c2 cells

**Fig. 3:**
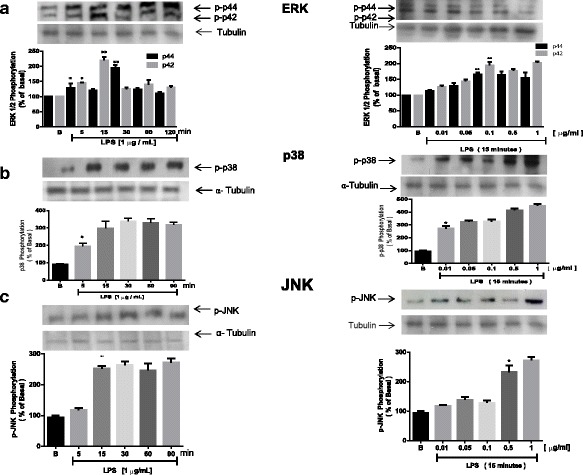
Lipopolysaccharide stimulation induces phosphorylation of ERK 1/2, p38, JNK in cardiomyoblast. H9c2 cells were incubated with LPS (1 μg/ml) for the indicated periods of time or with the indicated concentrations of LPS for 15 or 30 min. Cell lysates were separated by sodium dodecyl sulfate polyacrylamide gel electrophoresis (SDS-PAGE), transferred to a PVDF membrane, and immunoblotted with anti-phosphorylated-ERK 1/2 (**a**), −p38 (**b**) or -JNK antibodies (**c**). The membrane was then stripped and incubated with anti-tubulin antibodies. The data are representative of three independent experiments. The means ± SEM were obtained by densitometry. *p < 0.05 vs. untreated group

H9c2 cells were treated with LPS for the indicated periods and doses. We observed that LPS promoted phosphorylation of MAPK family members, such as ERK1/2 (a kinase activated by mitogens), p38 (a kinase activated by stress), and JNK. Maximal phosphorylation occurred 15 min post-treatment. For ERK, phosphorylation diminished at 60 min, possibly due to the activity of a phosphatase. Membranes were stripped and reprobed with anti-tubulin antibody to confirm equal loading in all lanes. To examine the direct effect of LPS on the activation of ERK1/2 (Fig. 3a), p38 (Fig. 3b) and JNK (Fig. 3c), H9c2 cells were treated with various concentrations of LPS (0.1–10 μg/ml) for 15 min to assess activation. As shown by western blotting (Fig. 3), LPS induced kinase phosphorylation in a dose-dependent manner. In addition, LPS induced kinase phosphorylation at concentrations as low as 0.1 μg/ml and reached a plateau at 5 μg/ml.

### Effect of flavonoids on LPS-induced MAPK phosphorylation in H9c2 cells

**Fig. 4:**
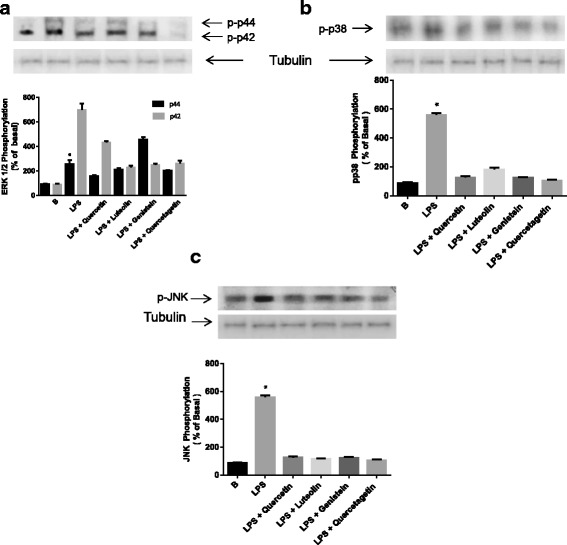
The effect of LPS on ERK 1/2, p38 and JNK phosphorylation in time- and dose-dependent response. H9c2 cells were placed in 6-well plates. Cells were stimulated with LPS (1 μg/ml) for the times and doses indicated in the figure. Cell lysates were fractionated via SDS-PAGE and analyzed via western Blotting using an antibody against phospho-ERK 1/2 (**a**), −p38 (**b**) or -JNK (**c**). Membranes were then stripped and reprobed using an antibody against each kinase as a control for equal loading. The data are representative of one of three independent experiments performed with similar results. Phosphorylation was quantified via densitometry with LabWorks 4.0 (Upland, CA, USA) commercial software. The results are expressed as means ± S.D. *p < 0.05 vs. untreated group

To determine the effect of flavonoids on LPS-induced ERK1/2 phosphorylation, cells were pre-incubated with the respective flavonoids (10 μM) for 1 h and then treated with LPS (1 μg/ml) for 15 min. Under these conditions, LPS promoted ERK1/2 phosphorylation (p44 and p42). Treatment with flavonoids blocked LPS-induced phosphorylation of ERK1/2. In addition, LPS-mediated ERK1/2 phosphorylation was completely blocked by quercetagetin and by luteolin (Fig. 4a).

We next tested the role of flavonoids in LPS-mediated inhibition of p38 phosphorylation. Treatment with luteolin and quercetagetin completely inhibited p38 phosphorylation, while genistein and quercetin inhibited p38 phosphorylation to a lesser extent (Fig. 4b).

Finally, we evaluated the effect of flavonoids on JNK phosphorylation. LPS induced a 5.5-fold increase in JNK phosphorylation above basal activity in H9c2 cells, while treatment with flavonoids completely inhibited JNK phosphorylation (Fig. 4c).

### Inhibition of LPS-induced degradation of IκB

**Fig. 5:**
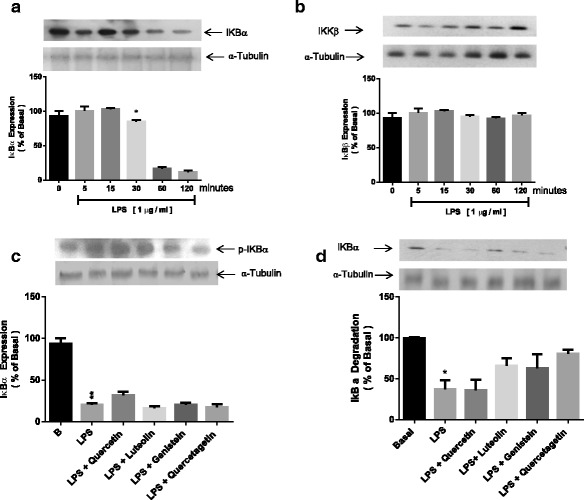
The effect of LPS on IkB phophorylation and degradation and flavonoids on IkB degradation. The time course of IκBα (**a**) and IκBβ degradation (**b**) and IκBα phosphorylation (**c**). Cells were pre-treated with flavonoids (10 μM) for 30 min and later exposed to LPS (1 μg/ml) for 15 min, to then be lysed. **d** – H9c2 cells were incubated with flavonoids (10 μM) for 30 min, then stimulated with LPS (1 μg/ml) for 60 min. Whole cell extract was processed via sodium dodecyl sulfate–polyacrylamide gel electrophoresis (SDS-PAGE), and the membranes were blotted with IκB-specific phosphoantibodies. The results are representative of three separate experiments. Densitometric analysis represents the means ± SEM. *p < 0.05 vs. untreated basal value

To determine whether flavonoids affected the LPS-induced degradation of IkB, we first established the levels of IκBα and IκBβ in H9c2 cells. We found that LPS (1 μg/ml) promotes IkBα degradation in a time-dependent fashion and IκBα decreased after 60 min of LPS incubation (Fig. 5a). Maximal phosphorylation was observed after 15 min of treatment. LPS did not show effect on IκBβ (Fig. 5b). Western blot analysis of cell extracts with IkBα-specific antibodies showed that treatment with flavonoids and LPS blocked this reduction in a concentration-dependent manner (Fig. 5c). Treatment with flavonoids blocked LPS-induced IkBα degradation. Luteolin significantly inhibited LPS-induced IκB degradation (Fig. 5d).

### Flavonoids reduce LPS-induced inflammatory COX-2 protein expression

**Fig. 6:**
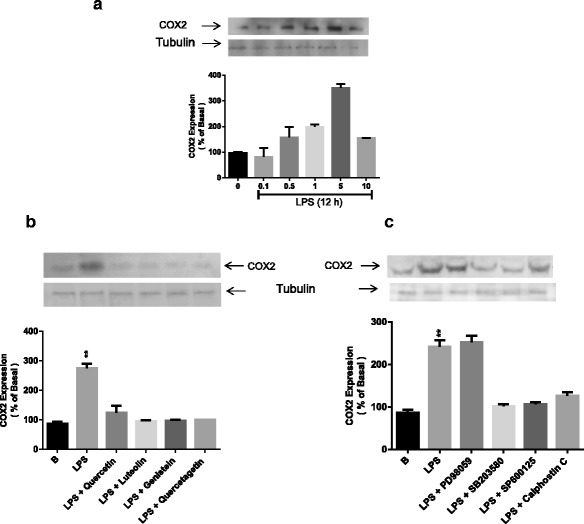
Effect of LPS and flavonoids on COX-2 expression. **a** – H9c2 cells were incubated with LPS at different doses. **b** – H9c2 cells were pretreated with flavonoids (10 μM) for 1 h and then incubated with LPS (1 μg/ml) for 12 h. **c** – H9c2 cells were pretreated with different inhibitors and then treated with LPS (1 μg/ml). The lysates of the cells were processed via sodium dodecyl sulfate–polyacrylamide gel electrophoresis (SDS-PAGE), and the membranes were blotted with COX-2-specific antibodies. The results are representative of three separate experiments. Tubulin was used as an internal control for western blot analysis. Statistical significance was determined with a two-way ANOVA test. *p < 0.05 vs. untreated group

We investigated the anti-inflammatory properties of flavonoids. During inflammation, large amounts of pro-inflammatory cytokines are synthesized. The western blot result showed that after 12 h treatment with LPS and flavonoids, these compounds significantly inhibited the expression of COX-2 induced by LPS (Fig. 6a). Furthermore, western blotting analysis showed that LPS treatment stimulated COX-2 expression, but that this LPS-induced expression was significantly downregulated by luteolin, genistein and quercetagetin. Quercetin was found to have no effect (Fig. 6b).

### p38 and JNK are involved in LPS-induced COX-2 expression

We evaluated the effect of inhibitors on COX-2 expression. Fig. 6c shows that LPS treatment stimulated COX-2 expression, which was significantly downregulated by SB203580 and SP600125. These data suggest that p38 and JNK are involved in COX-2 expression induced by LPS in H9c2 cells. However, ERK and PKC showed no effect on COX-2 expression. The results strongly suggest that p38 and JNK are involved in COX-2 expression.

## Discussion

This study demonstrated that LPS induced COX-2 expression in H9c2 cells, but that flavonoid pretreatment attenuated the inflammatory response and affected the MAPK pathway. The cardioprotective activity due to flavonoids downregulating COX-2 expression may be due to suppression of the MAPK signaling pathway. Chronic action of LPS disturbs the balance of tissue homeostasis, resulting in the accumulation of inflammatory response [23].

Flavonoids are widely found in the plant kingdom. Various studies have shown that they possess anti-inflammatory properties. Luteolin, genistein, quercetin and quercetagetin inhibited LPS-induced expression of inflammatory mediators such as TNFα and NO by suppressing NF-κB activation [22–24]. Furthermore, these flavonoids exerted inhibitory effects on IkB degradation.

In the LPS signaling pathways, four kinds of flavonoid may inhibit MAPK activation. Of the four flavonoids analyzed in this study, only the activity of luteolin had previously been reported on. In an experiment using human gingival fibroblasts, luteolin was shown to attenuate PGE2 and inhibit COX-2 gene expression. However, we found no effect of quercetin on COX-2 expression. We also reported that luteolin inhibited nuclear localization and transcriptional activation of NF-κB [24].

Xagogari et al. [12] also reported that luteolin inhibited IL-6- and TNF-α-mediated nuclear localization and transcriptional activation of NF-κB. All four kinds of flavonoid exert anti-inflammatory activity by inhibiting NF-κB activation in H9c2 stimulated with LPS.

Because LPS is an activator of IkB, we studied the inhibitory effects of these four flavonoids on LPS-induced IkB phosphorylation and degradation in H9c2 cells. A previous study reported that LPS promotes IκB-α phosphorylation and degradation. Here, we found that flavonoids blocked IκBα degradation and demonstrated that flavonoids strongly inhibited IκB-α activity in the cardiomyoblast cell line, H9c2. Some earlier reports [25–31] showed moderate inhibitory inflammatory activities for different flavonoids, but quercetin yielded the lowest level of inhibition.

Our investigation showed that flavonoids inhibit COX-2 production in LPS-treated H9c2 cells. The strongly active flavonoids possessed a C-2,3 double bond and 6-hydroxyl group in the A-ring, which suggests that the 6-hydroxyl moiety in the A-ring reduces COX-2 expression induced by LPS. For example, luteolin inhibits COX-2 expression to a greater degree than flavonol derivatives, such as quercetin.

## Conclusion

Our results show that flavonoids regulate the inflammatory response in cardiomyoblast. Of the assessed flavonoids, luteolin exhibited the best effects. Quercetagetin and genistein showed similar effects and quercetin had the lowest effect. Further studies might be needed to determine the target of luteolin in cardiomyoblast and evaluate its potential as a treatment for the cardiac inflammatory response.

## Abbreviations

AKT: Serine/threonine kinase
ANOVA: Analysis of variance
ATCC: American Type Culture Collection
COX-2: Cyclooxygenase-2
DMEM: Dulbecco’s modified Eagle’s medium
DMSO: Dimethyl sulfoxide
EDTA: Ethylene diamine tetraacetic acid
ERK: Extracellular signal-regulated kinases
FBS: Fetal bovine serum
GADPHP: Glyceraldehyde-3-phosphate dehydrogenase
IC50: Inhibitory concentration 50%
IkB: Inhibitor of kappa B
IL: Interleukin
JNK: c-Jun N-terminal kinases
LPS: Lipopolysaccharide
MAPK: Mitogen-activated protein kinase
MEK: MAPK kinase
MTT: 3-(4,5-dimethyldiazol-2-yl)-2,5-diphenyltetrazolium bromide
NF-κB: Nuclear factor kappa-light-chain-enhancer of activated B cells
NO: Nitric oxide
O.D.: Optical density
PAGE: Polyacrylamide gel electrophoresis
PBS: phosphate-buffered saline
Pg: *Porphyromonas gingivalis*
PKC: Protein kinase C
PMSF: Phenylmethylsulfonyl fluoride
PVDF: Polyvinylidene difluoride
SDS: Sodium dodecyl sulfate
SEM: Standard error of the mean
TLR4: Toll like receptor 4
TNF: Tumor necrosis factor
